# Case Report: Praziquantel-induced flare-up reaction in a rare case of diclofenac-induced probable DRESS comorbid with acute clonorchiasis: diagnostic and therapeutic challenges

**DOI:** 10.3389/fmed.2025.1678509

**Published:** 2025-10-28

**Authors:** Wei Li, Kaizhou Huang, Ying Chen, Yuping Huang, Yi Zheng, Minhua Zhong, Kaiping Jiang, Xiaojun Ma

**Affiliations:** ^1^The Eighth Clinical Medical College of Guangzhou University of Chinese Medicine, Foshan, China; ^2^Foshan Hospital of Traditional Chinese Medicine, Foshan, China; ^3^Chancheng District People's Hospital of Foshan City, Foshan, China

**Keywords:** clonorchiasis, praziquantel, flare-up reaction, drug reaction with eosinophilia and systemic symptom, multiple drug hypersensitivity

## Abstract

Drug reaction with eosinophilia and systemic symptoms (DRESS), a severe T-cell-mediated hypersensitivity with mortality up to 10%, may progress to life-threatening multi-organ failure and culminate in multiple drug hypersensitivity (MDH). Clonorchiasis, a hepatobiliary parasitic endemic in China, manifests with nonspecific symptoms including fever, jaundice, and abdominal discomfort. We report an unique case of diclofenac-induced probable DRESS comorbid with acute clonorchiasis in which a praziquantel (PZQ)-related flare-up reaction occurred in a 42-year-old male. Following praziquantel administration, the patient exacerbated skin lesions, acute liver/kidney failure, likely triggered by Clonorchis sinensis lysis-released antigens amplifying IgE-mediated responses and PZQ-induced hepatic injury. Despite the reaction onset exceeding PZQ's peak concentration timeline, a type IV hypersensitivity reaction to PZQ cannot be ruled out. Therapeutic intervention with plasmapheresis, continuous renal replacement therapy, intravenous immunoglobulin, and systemic corticosteroids achieved clinical stabilization. One year later, the patient developed isolated hepatitis following administration of a structurally unrelated nonsteroidal anti-inflammatory drug. Combined with previous medical history, MDH was highly suspected. This case underscores the diagnostic complexity in distinguishing parasitic infections from DRESS through parasitological confirmation, herpesvirus reactivation profiling, validated DRESS criteria, and lesional skin histopathology. Therapeutically, stepwise immunomodulatory prioritization for DRESS control is essential, with PZQ therapy restricted to life-threatening parasitosis only after achieving immune stability, under intensive monitoring for hypersensitivity recrudescence and end-organ damage.

## Context

Drug reaction with eosinophilia and systemic symptoms (DRESS) is a severe T cell-mediated hypersensitivity reaction characterized by widespread cutaneous eruptions, fever, and multi-organ involvement, predominantly affecting the liver and kidneys (1). The latency period between exposure and symptoms is usually long (2–6 weeks) and may persist for weeks after drug discontinuation (1). Flare-up reaction may occur with abrupt steroid tapering, new drug exposures, or herpesvirus reactivations (2). Clonorchiasis, caused by *Clonorchis sinensis* (C. sinensis), is mainly prevalent in Asian countries and regions, especially China, and manifests with nonspecific symptoms including fever, jaundice, and abdominal discomfort (3). We report an unique case of diclofenac-induced probable DRESS comorbid with acute clonorchiasis in which a praziquantel (PZQ)-related flare-up reaction occurred in a 42-year-old male. This culminated in acute hepatic/renal failure and sepsis, providing critical insights for clinical management of differential diagnosis and treatment.

## Case description

**Figure 1:**
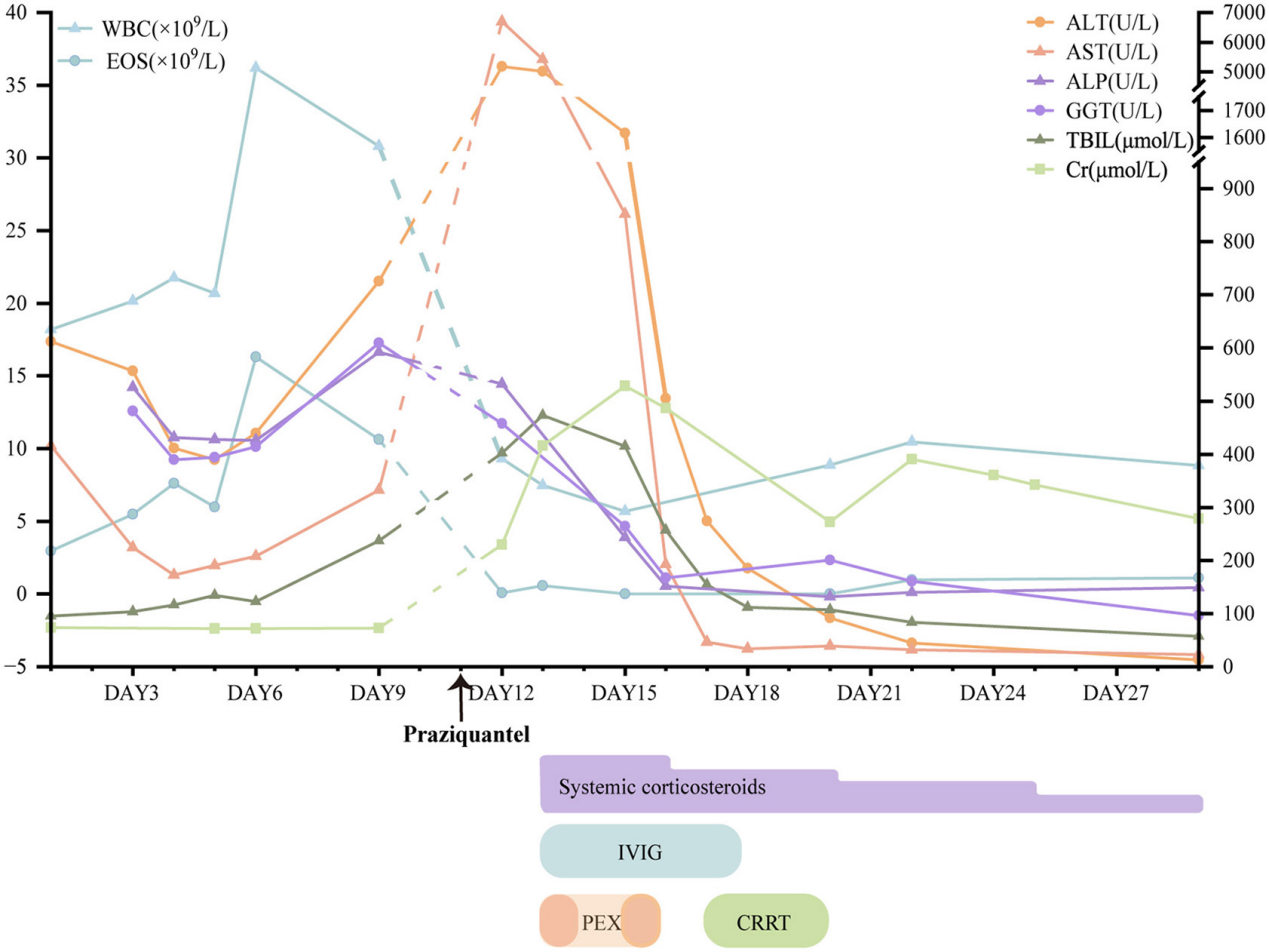
The results of partial laboratory studies and major treatment. Because laboratory results were not measured before and after praziquantel administration (the arrow), the dashed line was shown between Day 9 and 12 for unclear trend. WBC, white blood cells; EOS, eosinophil; AST, aspartate aminotransferase; ALT, alanine aminotransferase; ALP, alkaline phosphatase; GGT, gamma glutamyl transpeptidase; T-Bil, total bilirubin; Cr, creatinine; PEX, plasmapheresis; CRRT, continuous renal replacement therapy; IVIG, intravenous immunoglobulin.

A 42-year-old male presented to our hospital with a 4-day history of fever accompanied by abdominal distention, jaundice, and erythematous rash on the day of admission. One month ago, he had taken diclofenac several times for low back pain. He denied previous drug allergies, and his medical history included raw freshwater fish consumption. Bilateral lymphadenopathy with the largest nodes measuring approximately 1.5 cm in diameter was palpable in the neck and submandibular angle. Admission laboratory studies revealed leukocytosis (18.21 × 10^9^/L; normal range [NR]: 3.5–9.5 × 10^9^/L) with marked eosinophilia (2.97 × 10^9^/L; NR: 0.02–0.52 × 10^9^/L). Hepatic dysfunction was evident in aspartate aminotransferase (AST) 415.8 U/L (NR: 15–40), alanine aminotransferase (ALT) 611.9 U/L (NR: 9–50), and total bilirubin 95 μmol/L (Figure 1). Viral hepatitis serologies, autoimmune hepatitis markers, and hemoculture were negative. Abdominal CT demonstrated cholecystitis, peritonitis, subcapsular and right abdominal effusion, and multiple enlarged lymph nodes in the hilar region and retroperitoneum. With an initial diagnosis of acute cholecystitis, he received ultrasound-guided gallbladder drainage and injection of diammonium glycyrrhizinate and adenosylmethionine butanedisulfonate. On day 4, the patient developed high-grade fever (39.5 °C) with a generalized erythematous rash and papular lesions involving >50% of body surface area, distributed across the extremities, trunk, and palms. The clinical presentation was interpreted as acute cholecystitis comorbid with diclofenac-induced cutaneous adverse reaction. Therapeutic interventions included oral methylprednisolone (12 mg/day) and intravenous dexamethasone (5 mg/day) to mitigate hypersensitivity manifestations.

**Figure 2:**
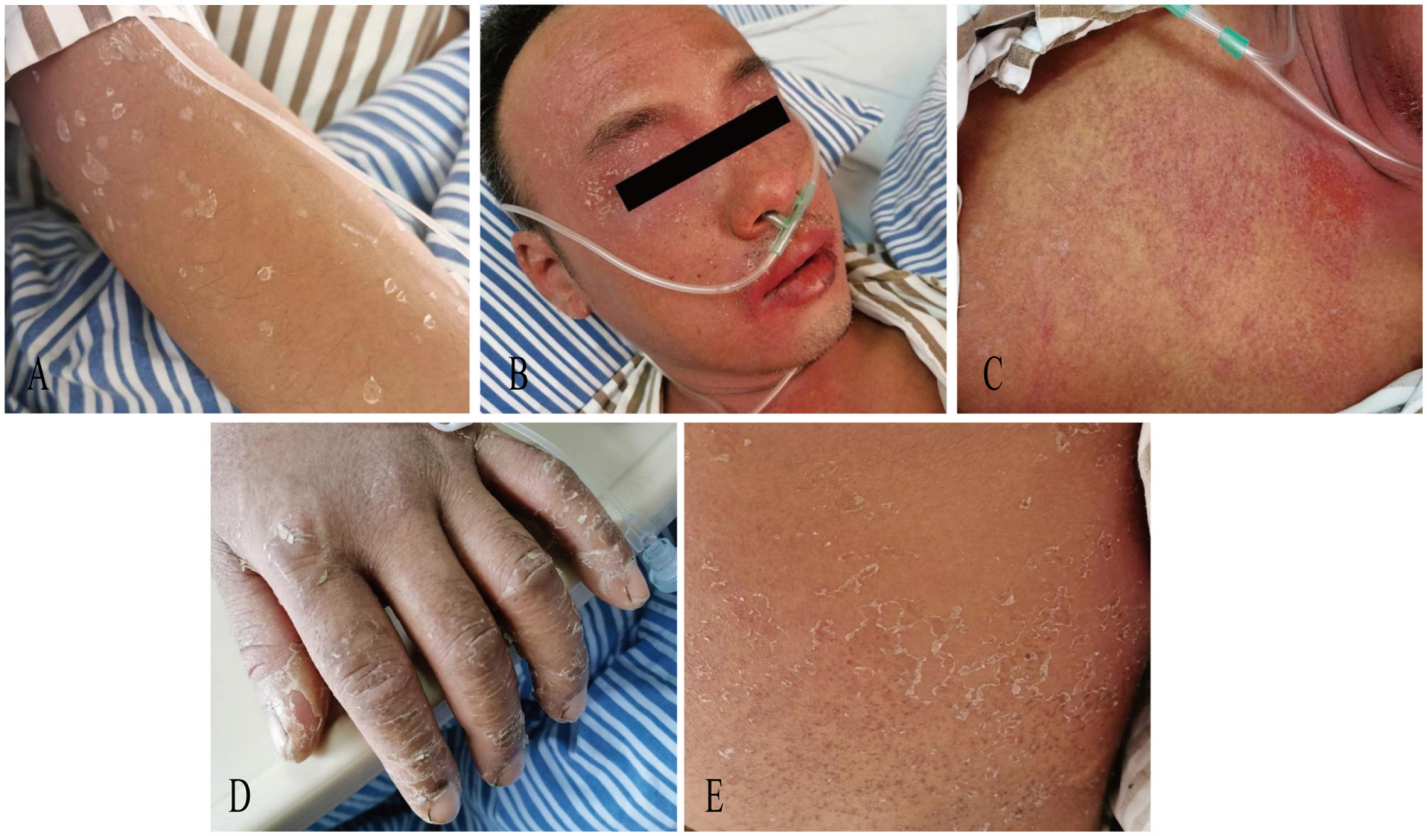
Photograph of scattered desquamation **(A)** on Day11, orofacial angioedema with perioral erosions **(B)** and a generalized erythematous rash **(C)** on Day12, desquamation with hyperpigmentation **(D)** and truncal pinpoint papules **(E)** on Day 19.

On day 11, the patient exhibited partial resolution of cutaneous manifestations with generalized scattered desquamation (Figure 2A), though persistent fever and scleral icterus remained. Laboratory investigations revealed marked elevation of hepatic enzymes, eosinophilia, and hyper-immunoglobulinemia E (>2,500 IU/mL). Morphological abnormalities of peripheral blood cells showed 44% eosinophil and 2% atypical lymphocyte. Bile microscopy revealed numerous Clonorchis sinensis eggs. Negative serology for *Paragonimus westermani, Angiostrongylus cantonensis, Schistosoma japonicum*, and *Plasmodium spp*. IgG antibodies. A definitive diagnosis of clonorchiasis was established, prompting initiation of PZQ (total dose 210 mg/kg, 3 times a day for 3 days).

One day later, the patient developed a generalized erythematous rash with orofacial angioedema, perioral erosions, xerostomia, oliguria, and progressive jaundice (Figures 2B, C). Hepatorenal dysfunction demonstrated ALT of 5179 U/L (NR: 9–50), AST of 6,689.3 U/L (NR: 15–40), and total bilirubin of 402.6 μmol/L. The creatinine was 229.4 μmol/L. Systemic inflammation revealed C-reactive protein of 148 mg/L and procalcitonin of 2.3 ng/mL. The human herpesvirus-6 (HHV-6) and Epstein-Barr virus (EBV) serology were negative. The clinical constellation met RegiSCAR criteria for probable DRESS (Table 1) (4), with concurrent acute liver/kidney failure, and sepsis (Sequential Organ Failure Assessment (SOFA) score of 5 points). Therapeutic interventions included plasmapheresis (PEX) for 2 sessions, continuous renal replacement therapy (CRRT) for 40.9 h, intravenous immunoglobulin (IVIG) 50 ml for 6 days, and IV intravenous methylprednisolone (80 mg/day for 3 days, tapered to 40 mg for 4 days). On day 19, cutaneous findings evolved to generalized desquamation with hyperpigmentation and truncal pinpoint papules (Figures 2D, E). Oral Prednisolone tapering occurred gradually with improvement of symptoms and laboratory tests for three weeks.

**Table 1:** RegiSCAR scoring for DRESS syndrome (4).

**RegiSCAR group study criteria**	**Yes**	**No**	**Unknown**	**Before**	**After**
Fever ≥ 38.5 °C	0	−1	0	0	0
Enlarged lymph nodes (≥2 sites, >1 cm)	1	0	0	1	1
Eosinophilia	0	0	0	0^*^	0^*^
0.7–1.499 × 10^9^/L (or 10%−19.9% if leukocytes < 4.0 × 10^9^/L)	1				
≥1.5 × 10^9^/L (or ≥ 20% if leukocytes < 4.0 × 10^9^/L)	2				
Atypical lymphocytes	1	0	0	1	1
Skin rash extent >50% BSA	1	0	0	1	1
At least 2 of: edema, infiltration, purpura, scaling	1	−1	0	1	1
Biopsy suggesting DRESS	0	−1	0	−1	−1
Internal organ involved		0	0	0^*^	1^*^
1	1				
≥2	2				
Resolution ≥15 days	0	−1	1	1	1
Evaluation of other potential causes	1	0	0	0	0
Total				4	5

Before: before PZQ administration; After: after PZQ administration; Total: 2 to 3 (possible case), 4 to 5 (probable case), and > 5 (definitive case); ^*^Points for eosinophilia and hepatic involvement were not assigned, as these findings could be attributed to either DRESS syndrome or the concomitant acute clonorchiasis. A conservative scoring approach was adopted, classifying them as “unknown” etiology.

One year later, he was hospitalized again because of drug-induced liver injury after taking nimesulide and dexketoprofen tromethamine tablets. Laboratory studies revealed ALT of 3,056.7 U/L, AST of 2,178.1 U/L, eosinophilia of 17.5%, and immunoglobulin E (IgE) of 839.2IU/ml with elevated tumor necrosis factor α and interleukin 6. Following injection of magnesium isoglycyrrhizinate and oral prednisone acetate tablets, the patient's condition improved. Although the patient did not present with hypersensitivity-associated skin eruptions during the latter hospital stay, we suspect multiple drug hypersensitivity (MDH) triggered by structurally unrelated NSAIDs based on clinical history. The final hospitalization manifested as isolated drug-induced liver injury. We recommended further diagnostic investigations including Patch Tests and Lymphocyte Transformation Test (LTT) to confirm MDH, but the patient declined. Consequently, we strongly advise patients to avoid NSAIDs whenever possible. If NSAID use is unavoidable, high-affinity NSAIDs at minimal effective doses such as celecoxib or etoricoxib should be prioritized.

## Discussion

This case highlights the importance of early DRESS recognition and the risk of using PZQ in patients with hypersensitivity reactions. Given the significant overlap in laboratory (elevated eosinophils, hepatic dysfunction, and hyper-IgE) and clinical presentations between clonorchiasis and DRESS, we calculated the RegiSCAR score conservatively. Although marked eosinophilia and hepatic dysfunction are classic for DRESS, these parameters were classified as “unknown” regarding their etiology, and points were not awarded for them in the scoring system. Despite this, the total score still met the threshold for a “probable” case of DRESS, underscoring the strength of the clinical diagnosis even amidst significant diagnostic challenges. In this case, clonorchiasis was confirmed through microscopic identification of *C. sinensis* eggs in both stool and bile specimens. While low-dose corticosteroids therapy led to partial resolution of cutaneous manifestations, the rarity and variability of DRESS likely contributed to the clinicians' hesitation in pursuing further investigations for severe drug hypersensitivity or alternative etiologies. Pathophysiologically, clonorchiasis primarily manifests through direct mechanical obstruction and inflammatory infiltration of bile ducts by adult flukes, typically resulting in obstructive jaundice. This case exhibited mixed-type liver injury (R ratio[ALT/ULN ÷ ALP/ULN] of 2–5) rather than pure cholestasis, accompanied by progressive hepatic synthetic decline evidenced by hypoalbuminemia and coagulopathy. In addition, the combination of markedly elevated levels of high-sensitivity C-reactive protein, eosinophils, and the extent of body surface area involvement can be used for early DRESS recognition (5). Furthermore, classic features such as facial edema and the presence of atypical lymphocytes are also pivotal clues for diagnosis (4).

The prevailing hypothesis on the pathogenesis of DRESS posits a multifactorial interplay between drug-specific immune activation and dysregulated antiviral responses (6). When exposed to a drug, its metabolites with T-cell receptors and human leukocyte antigen complexes bypassing any prior metabolic processing or protein binding to initiate T-cell clonal expansion, thereby escalating hypersensitivity reactions. The cytokines such as IL-4, IL-5, and IL-13 are released causing eosinophilia and the pro-inflammatory cytokines like IFN-γ, TNF, IL-6, and IL-15 are increased promoting systemic inflammation. After PZQ administration, the patient experienced flare-up reaction of DRESS, characterized by exacerbated cutaneous manifestations, acute liver failure, and acute kidney failure. In this patients, the HSV and EBV serology of the patients were negative. Critically, the abrupt lysis of Clonorchis sinensis may release parasite derived antigens following PZQ administration, which may act as pathogen-associated molecular patterns to amplify mast cell degranulation and IgE-mediated anaphylactoid responses (7). Furthermore, PZQ is mainly metabolized in the liver and may aggravate drug-induced hepatic injury. Notably, the time for flare-up reaction has already exceeded the time when PZQ reached its maximum concentration, but a type IV hypersensitivity reaction to PZQ cannot be ruled out. Unfortunately, the patient did not consent to the lymphocyte transformation test. Combination therapy with systemic glucocorticoids, PEX, CRRT, and IVIG achieved clinical stabilization. This antigenic surge, coupled with pre-existing drug hypersensitivity, likely synergized to potentiate multi-organ involvement in this patient.

## Conclusion

To our knowledge, the first reported case of DRESS comorbid with clonorchiasis highlights two critical imperatives: (1) Systematic diagnostic differentiation through parasitological confirmation (microscopy/serology), herpesvirus reactivation profiling, validated DRESS criteria (RegiSCAR), and lesional skin histopathology; (2) Stepwise immunomodulatory prioritization for DRESS control, restricting PZQ therapy to life-threatening parasitosis only after achieving immune stability, with intensive monitoring for hypersensitivity recrudescence and end-organ damage.

## Data Availability

The original contributions presented in the study are included in the article/supplementary material, further inquiries can be directed to the corresponding authors.
